# One Stone, Two Birds: The Roles of Tim-3 in Acute Myeloid Leukemia

**DOI:** 10.3389/fimmu.2021.618710

**Published:** 2021-04-01

**Authors:** Zhiding Wang, Jinghong Chen, Mengzhen Wang, Linlin Zhang, Li Yu

**Affiliations:** ^1^ Department of Hematology and Oncology, International Cancer Center, Shenzhen Key Laboratory of Precision Medicine for Hematological Malignancies, Shenzhen University General Hospital, Shenzhen University Clinical Medical Academy, Shenzhen University Health Science Center, Shenzhen, China; ^2^ Beijing Institute of Basic Medical Sciences, Beijing, China; ^3^ Department of Hematology and BMT Center, Chinese PLA General Hospital, Beijing, China

**Keywords:** Tim-3, AML, LSC, immunotherapy, antibody

## Abstract

T cell immunoglobulin and mucin protein 3 (Tim-3) is an immune checkpoint and plays a vital role in immune responses during acute myeloid leukemia (AML). Targeting Tim-3 kills two birds with one stone by balancing the immune system and eliminating leukemia stem cells (LSCs) in AML. These functions make Tim-3 a potential target for curing AML. This review mainly discusses the roles of Tim-3 in the immune system in AML and as an AML LSC marker, which sheds new light on the role of Tim-3 in AML immunotherapy.

## Introduction

T cell immunoglobulin and mucin protein 3 (Tim-3) was first discovered in 2002 and is a type I membrane-bound glycoprotein (1). Tim-3 belongs to the Tim domain gene family of immunoregulatory proteins and plays a role in biological responses in human immune cells. The human gene HAVCR2 encodes Tim-3 and is located on chromosome 5q33.2 (2). The genomic region where the HAVCR2 gene is located is correlated with asthma and allergies and is near the centromeric end of the IL-4, IL-5, and IL-13 gene loci (2, 3). The structure of Tim-3 is composed of an amino-terminal immunoglobulin variable domain (V domain) with five noncanonical cysteines, a mucin stalk, a transmembrane domain, and a cytoplasmic tail (2). Targeting Tim-3 could balance the immune system and kill LSCs, which may be a potential AML therapeutic strategy.

## Tim-3 and the Immune System

Tim-3 plays a vital role in immune tolerance. Tim-3 was originally identified as being expressed on IFN-γ-producing CD4+ and CD8+ T cells, and Tim-3 is now known to be expressed on Treg cells (4), macrophages (5), natural killer (NK) cells (6), dendritic cells (DCs) (7), mast cells (8) and other lymphocyte subsets. Recent studies have shown that Tim-3 is involved in immune suppression in both the innate and adaptive immune systems (9). Thus, targeting Tim-3 on multiple types of immune cells might improve the efficacy of cancer immunotherapy.

### Tim-3 Is a Marker of Dysfunctional T Cells

Tim-3 is used as an important surface marker for exhausted and dysfunctional T cells (9). Some studies have shown that Tim-3 is part of a network that contains multiple checkpoint receptors that are coexpressed and coregulated on dysfunctional or ‘exhausted’ T cells during chronic viral infections and cancers (10, 11). Studies have indicated that Tim-3-expressing CD4^+^ T cells in human tumors could define the functional regulatory T cells that contribute to the immunosuppressive tumor micromilieu (12). Tim-3 is also involved in Th1-dependent immune responses and induces immune tolerance (1).

### Tim-3 Negatively Regulated Macrophages Function

Studies have also shown that Tim-3 negatively regulates macrophage function. Tim-3 blockade was shown to enhance macrophage function in response to sepsis (13). Wang et al. (14) demonstrated that Tim-3 inhibited macrophage phagocytosis of *Listeria monocytogenes* by inhibiting the Nrf2-CD36 signaling pathway. Recently, Wang et al. (15) also found a new mechanism by which Tim-3 promoted *L. monocytogenes* immune evasion by decreasing macrophage MHC-I antigen presentation. However, the function of Tim-3 in macrophages is still unclear.

### Tim-3 Is a Benchmark for NK cell Dysfunction

Tim-3 has been identified as a benchmark for human NK cell dysfunction (6). Downregulated Tim-3 expression in NK cells showed that NK cell-mediated cytotoxicity was inhibited and IFN-γ production was decreased in tumors and leukemia (16–18). IFN-γ can impair NK cell-mediated cytotoxicity by inducing the activation of indoleamine 2,3-dioxygenase (IDO1) in AML (17). Tim-3 is involved in the dysfunction of both tumor-infiltrating liver-resident and conventional NK cells by disrupting PI3K signaling, thereby enhancing hepatocellular carcinoma growth (19).

### Tim-3 Inhibits DCs Function

Tim-3 is highly expressed on tumor-associated DCs in mouse tumors and patients with cancer (20). An anti-Tim-3 antibody improved the response to chemotherapy in a mouse breast cancer model and increased CXCR3 chemokine ligand CXCL9 expression by tumor DCs. Nucleic acid-mediated innate immune responses can be suppressed by DC-derived Tim-3 through Toll-like receptors *via* a galectin-9-independent mechanism (21). Moreover, Tim-3 can interact with high-mobility group protein B1 (HMGB1) to interfere with the recruitment of nucleic acids into DC endosomes and attenuate the therapeutic efficacy of DNA vaccination and chemotherapy by diminishing the immunogenicity of nucleic acids released from dying tumor cells.

### Tim-3 May Have Activating Function in Mast Cells

Tim-3 mediates the activation of mast cells, in contrast to its inhibitory effects in T cells. It was reported that mast cells constitutively express Tim-3 on the cell surface and that Tim-3 could enhance cytokine production in IgE-sensitized and Ag-stimulated BM-derived mast cells (BMMCs) and peritoneal mast cells (pMCs) without affecting degranulation (22). The production of IL-3, IL-4, IL-6, and IL-13 in mast cells is enhanced by Tim-3 antibodies following antigen-dependent activation and IgE (FcϵRI) sensitization in mast cells *in vitro (* 8). Tim-3 can enhance FcϵRI-proximal signaling and increase cytokine production downstream (23). Although previous data have suggested that Tim-3 is a positive regulator of mast cell activation, the molecular mechanisms by which Tim-3 affects mast cell function are still unknown.

## Tim-3 and Its Ligands

**Figure 1:**
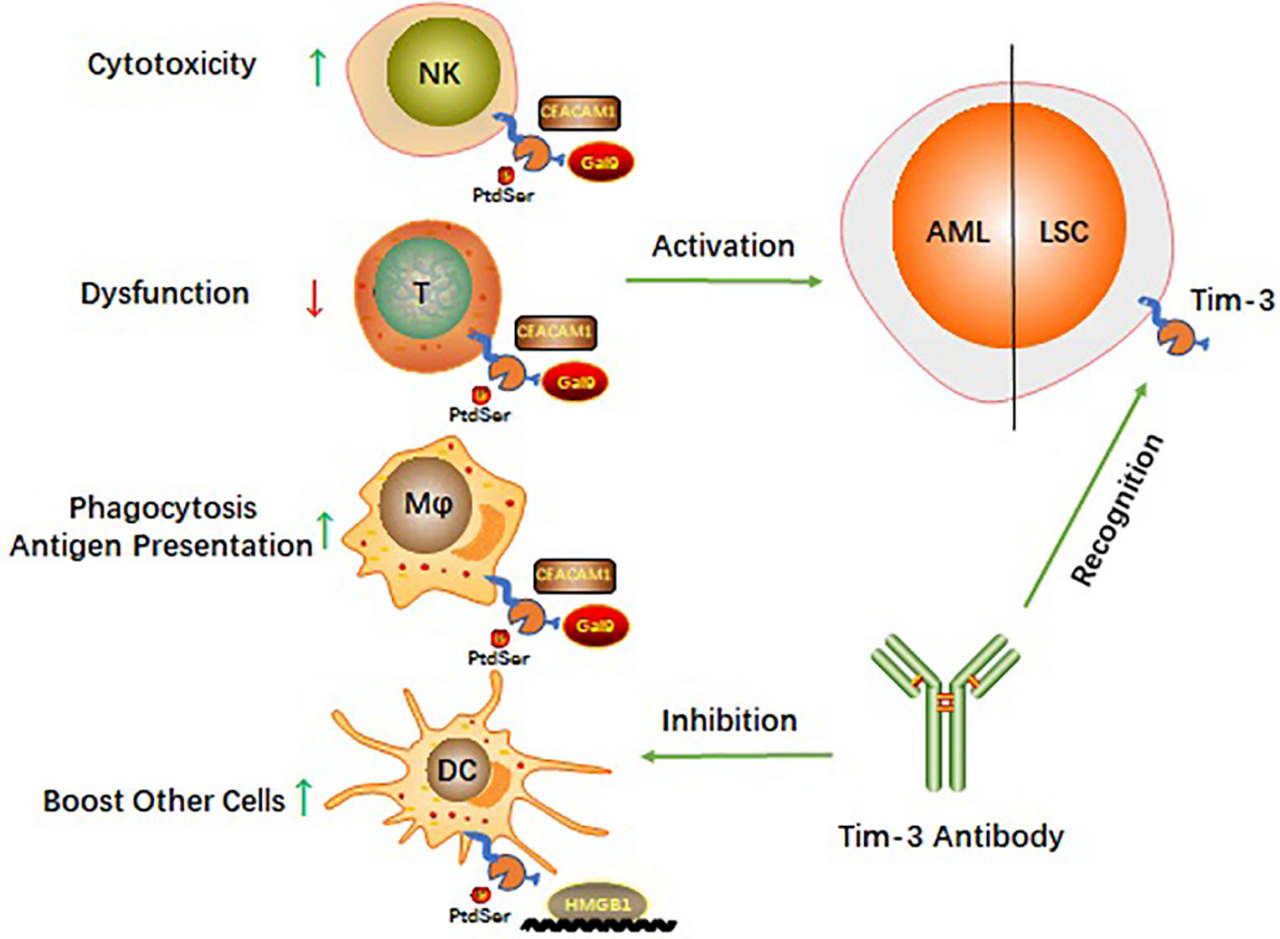
Schematic diagram of Tim-3 roles in AML.

Four relevant ligands have been reported to interact with different regions of the Tim-3 extracellular immunoglobulin V domain (9). These include galectin 9 (Gal-9), phosphatidylserine (PtdSer), HMGB1, and cell adhesion molecule bound to carcinoembryonic antigen 1 (CEACAM1). How Tim-3 interacts with each of these ligands (**Figure 1**) and the biological consequences of these interactions are described in the following subsections.

## Gal-9

Gal-9 was identified as a natural ligand of Tim-3. Gal-9 contains two distinct carbohydrate receptor-binding domains (24) and can form an autocrine loop with Tim-3, which is a positive feedback interaction (25). Multiple lines of evidence highlight the role of the Tim-3/Gal-9 interaction in mediating the inhibition of immune responses in different cell types (9).

## Phosphatidylserine

The second Tim-3 ligand identified is PtdSer, which is released from apoptotic cells (26). PtdSer was shown to bind the FG–CC’ cleft site of Tim-3 (27). PtdSer and Tim-3 binding contributes to the clearance of apoptotic bodies and antigen cross-presentation by Tim-3^+^ DCs (7). Phosphorylation of Tim-3 is facilitated by PtdSer engagement and results in the dysfunction of both tumor-infiltrating liver-resident and conventional NK cells by disrupting PI3K signaling (19).

## HMGB1

HMGB1 is the ligand of Tim-3 (28) and can be secreted by dead tumor cells (26). Tim-3 is highly expressed on tumor-infiltrating DCs and may act as a molecular sink of the alarmin HMGB1, with the recruitment of nucleic acids released from dying tumor cells, but the binding site of the Tim-3/HMGB1 interaction has not been identified (26, 29). Stimulation of the innate immune response to nucleic acids can be suppressed due to the binding of Tim-3 and HMGB1 on DCs in the tumor environment (19).

## CEACAM1

A more recently identified ligand of Tim-3 is CEACAM1, which is expressed on the surface of DCs, monocytes, macrophages (30), and activated T cells (31). CEACAM1 can bind to the CC’ and FG loops of Tim-3 and form a specific heterodimer in cis or in trans (31–33). Both cis and trans interactions between CEACAM1 and Tim-3 determine T cell immune tolerance. CEACAM1/Tim-3 complex formation has a crucial role in regulating autoimmunity and antitumor immunity (31). However, CEACAM1 may also have a tumor-suppressive function under some circumstances, since it was found that CEACAM1^−/−^ mice developed a higher tumor burden than wild-type mice. Therefore, the Tim-3/CEACAM1 interaction in the tumor microenvironment is very complex and further exploration of the underlying molecular mechanisms is needed to elucidate the regulatory mechanism (31).

## Roles of Tim-3 in the Immune Response in AML

AML is a malignant disorder of hemopoietic stem cells (34). Scientists have found that AML cells can evade immune attack after exposure to immune cells (35, 36). Several important biochemical mechanisms allow AML cells to escape from immunological synapses of cytotoxic lymphoid cells and comprehensively inactivate anticancer immunity from a distance. AML cells are capable of escaping immune attack, even though these cells are frequently exposed to host immune cells. In this context, AML cells not only “fight back” against immune cells but also effectively prevent the process of cytotoxic immune attack (37, 38). IL-2 expression is significantly lower in blood samples from AML patients than in blood samples from healthy individuals (36). Soluble Tim-3 in AML could inactivate cytotoxic lymphoid cells by downregulating IL-2 expression (37). Tim-3 is a coinhibitory receptor that is expressed on IFN-γ-producing T cells, FoxP3^+^ Treg cells, and innate immune cells, where Tim-3 has been shown to suppress immune cell responses *via* ligand interactions. Tim-3 has gained prominence as a potential candidate for cancer immunotherapy, and *in vivo* blockade of Tim-3 with other checkpoint receptors enhances antitumor immunity and suppresses tumor growth in several preclinical tumor models (39).

Gal-9 in cytotoxic lymphoid cells is capable of impairing anticancer activities (25). The Gal-9/Tim-3 interaction is capable of activating downstream signaling pathways such as the transcription factor NF-κB (40) to support the survival of AML cells. Mammalian target of rapamycin (mTOR) controls translation-related pathways, which are also downstream signaling pathways of Tim-3 (41, 42). Prokhorov et al. (42) showed that Gal-9/Tim-3 can activate the phosphatidylinositol 3 kinase (PI3K)/mTOR signaling pathway to support growth factor responses in AML. Moreover, Gal-9/Tim-3 can activate hypoxic signaling, which can increase glycolysis and strengthen the proangiogenic response. Hypoxic signaling pathways are generally essential for AML cells to adapt to stressful conditions for survival (36, 42).

Gal-9 can bind to the extracellular domain of the Tim-3 receptor in humans, and Gal-9 levels are increased in AML patient serum (43). Silva et al. (25) first showed that Tim-3 was essential for the secretion of Gal-9 in some human AML cell types. This team proposed that there was a positive feedback interaction between Gal-9 secretion and Tim-3 expression (25). Cytotoxic lymphoid cells in AML could be suppressed from a distance by AML cells after the formation of a Tim-3 and Gal-9 autocrine loop. This indirect interaction could contribute to AML cell self-renewal, resulting in the rapid development of AML (25). Additionally, the exocytosis of Tim-3 and Gal-9 can be facilitated by the neuronal receptor latrophilin 1 (LPHN1), which contributes to cell survival. LPHN1 still appears in AML but generally disappears after maturation in hematopoietic stem cells (HSCs) (25).

Stem cell factor (SCF) is a key cytokine that contributes to the development of AML, and it is important to understand the mechanism underlying the interactions of Tim-3-mediated responses with SCF-induced signaling networks (44–46). Prokhorov et al. (42) first showed that the effects of high-affinity antibody-mediated activation of Tim-3 were similar to those of SCF activation, while SCF-dependent responses were not found to be effectively potentiated by any of the investigated ligands. It is important that SCF-dependent signaling pathways can induce proinflammatory responses to change the expression of Tim-3.

Studies relating to Tim-3 function in AML are limited, and some studies have also shown a correlation between Tim-3 and AML. Tim-3 levels were higher in AML than in other AML cytogenetic subgroups and associated with core-binding factor translocations or mutations in CEBPA (47). Researchers found that the activation of monocyte-associated genes was correlated with Tim-3 expression in AML. Other studies have shown that miRNAs are capable of affecting AML suppression or progression through gene expression. For example, Tim-3 expression could be inhibited by miR-330-5p (48), miR-125a-3p (49) and miR-498 (50). However, the underlying mechanism by which Tim-3 is involved in immune responses in AML is not fully understood.

## Tim-3 as an LSC Marker in AML

Immature leukemic blast cells are largely produced by leukemic progenitors, which are generated by self-renewing LSCs in the bone marrow (51–53). Approximately 90% of AML patients achieve complete remission after intensive chemotherapy, but approximately 60% of those patients eventually relapse (28). Relapse and refractoriness in AML are mainly due to residual LSCs that are capable of regrowth. Thus, the eradication of LSCs might be a therapeutic strategy (54). Little is known about the signaling mechanisms underlying LSC self-renewal (40). Hematopoietic tissues in AML patients contain both LSCs and residual normal HSCs (47).

The two most commonly used markers to distinguish and select LSCs are CD34 and CD38, which have different expression levels in different fractions, making it difficult to select LSCs. CD44, CD25, CLL-1, CD32, CD96, CD47, CD70 and CD123 were found to be preferentially expressed on AML LSCs. However, the expression levels of some of them are insufficient for selection (5). Recent studies have found that CD70 and CD47 antibodies have therapeutic functions in AML in clinical trials. Riether et al. (55) found that targeting CD70-expressing LSCs with the antibody cusatuzumab with enhanced antibody-dependent cellular cytotoxicity (ADCC) activity can eliminate LSCs *in vitro* and *in vivo*. A phase 1/2 trial in previously untreated older patients with AML investigated a single dose of cusatuzumab monotherapy with azacitidine (NCT03030612). The hematological responses in the 12 patients enrolled included 8 complete remission, 2 complete remission with incomplete blood count recovery and 2 partial remission, with 4 patients achieving minimal residual disease negativity. No dose-limiting toxicities were reported. Treatment with the CD70 antibody cusatuzumab substantially reduced the number of LSCs and triggered gene signatures related to myeloid differentiation and apoptosis. Sallman et al. (56) found that the CD47 antibody magolimab (Hu5F9-G4) with azacytidine (NCT03248479) therapy achieved hematological responses in 16 of the 25 enrolled patients, including 10 patients with complete remission, 4 patients complete remission with incomplete blood count recovery and 1 patient with partial remission.

Tim-3 is a potential therapeutic marker of LSCs in AML. Tim-3 is a target for selectively killing LSCs but not HSCs in most human AML cells. A study by Hope et al. (52) revealed a significant association between the aggressiveness of acute myeloid leukemia and Tim-3 levels in the Kasumi-1 and KG-1a cell lines. Jan et al. (57) reported that multistep leukemogenesis occurs from self-renewing HSCs by analyzing somatic mutations and found that Tim-3 is a possible and useful target for eradicating LSCs and sparing residual HSCs. Kikushige et al. (51) revealed that LSCs in CD34^+^CD38^-^ AML could functionally express the Tim-3 protein, while this expression did not occur in normal HSCs or myeloerythroid or lymphoid progenitor populations. Jan et al. (47) reported a finding similar to that of Kikushige et al. Tim-3 could be used to separate LSCs from normal HSCs and is a potential marker for LSC-targeted monoclonal antibodies in AML patients. Recently, Haubner et al. (58) found that LSCs in 78.5% of 302 AML patients were positive for Tim-3 at initial diagnosis. These studies indicated that normal hematopoiesis might not be seriously affected by the use of Tim-3 as a marker to target LSCs in AML.

Tim-3 plays a vital role in AML LSCs in AML. As cytokines or growth factors can be produced by myeloid leukemia cells, AML cells are capable of stimulating their own expansion for survival (59). The Tim-3/Gal-9 autocrine loop plays a key role in the self-renewal of LSCs and the maintenance of AML (40). Elevated levels of Gal-9 have been detected in the serum of AML patients and the serum of xenografted models reconstituted with human AML samples. The NF-κB pathway is constitutively active in LSCs, which is not the case in normal HSCs (59). The mechanisms underlying the activation of NF-κB in myeloid leukemias might partly be caused by the formation of the Tim-3/Gal-9 autocrine loop (40). β-Catenin is an important protein in the self-renewing organization of both normal and malignant hematopoietic stem cells (60). Gal-9 could stimulate Tim-3 and then coactivate the β-catenin pathway, which plays a role in supporting the self-renewal of LSCs (40). Therefore, it is important to draw attention to the therapeutic strategies associated with the Tim-3/Gal-9 autocrine loop (40). Additionally, the survival and proliferation of LSCs can be promoted by tumor-associated macrophages (TAMs), which differentiate from myeloid-derived suppressor cells (MDSCs). Expanded MDSCs could induce Tim-3 expression on T cells to suppress immunity. Understanding the correlation between TAMs in AML and the Tim-3/Gal-9 interaction could be useful for eradicating LSCs (5).

Tim-3 is a potential therapeutic marker of LSCs in AML. The role of Tim-3 in maintaining LSCs and contributing to the suppression of the antitumor immune response is still poorly understood. The potential therapeutic function and mechanism of Tim-3 in LSCs in AML remain to be explored.

## Tim-3 as a Therapeutic Target

### Preclinical Research

Some studies have shown the therapeutic potential of Tim-3 in AML preclinical research. Tan et al. (61) revealed that the leukemic immunosuppressive microenvironment was affected by the upregulation of PD-1 and Tim-3 in exhausted CD4^+^ and CD8^+^ T cells in the bone marrow in 15 AML patients. Wu et al. (62) showed that Vδ2 T cell production of TNF-α and IFN-γ was increased by Tim-3 inhibition in combination with PD-1 inhibition but was insufficiently affected by PD-1 inhibition alone in AML patients. These findings revealed that Tim-3 expression could be significantly upregulated in Vδ2 T cells after the administration of anti-PD-1 antibodies, which indicates that PD-1 inhibition alone is not able to activate Vδ2 T cells (62). Proliferation and effector functions can be impaired by combining the Tim-3 and PD-1 signaling pathways in exhausted T cells (63). PD-1 is expressed on the surface of these exhausted T cells. Blocking the PD-1/PD-L1 checkpoints can contribute to the functional revival of T cells in mice (64). In preclinical models, Tim-3 blockade in combination with PD-1 is capable of improving antitumor immunity and contributes to tumor regression. Additionally, some studies have revealed that anti-cancer response-related vaccines and chemotherapy are negatively affected by the upregulation of Tim-3 on tumor-associated DCs and macrophages (65). Cancer vaccines in combination with anti-Tim-3 mAbs could be used as an effective therapeutic strategy, which suggests an interesting new strategy for AML immunotherapy (66, 67).

Anti-Tim-3 antibodies can reduce the effect of LSCs, which may be a practical approach to curing human AML (65). Jan et al. (47) used xenograft mouse models infused with CD34^+^CD38^-^Tim-3^-^ and CD34^+^CD38^-^Tim-3^+^ cells from AML patients, and only CD34^+^CD38^-^Tim-3^+^ cells could support reconstitution and transplant leukemic engraftment, which indicated that Tim-3 could be used as a marker to distinguish residual HSCs from LSCs in AML. Kikushige et al. (51, 68) used xenograft models and found that ATIK2a, an anti-Tim-3 antibody, reduced CD34^+^CD38^–^ LSC numbers and eliminated functional LSCs in primary recipients. ATIK2a successfully killed Tim-3-expressing cell lines *via* both complement-dependent cytotoxicity and antibody-dependent cellular cytotoxicity effects (51). Notably, ATIK2a did not harm reconstituted human HSCs or human hematopoiesis *in vivo*. The researchers identified ATIK2a as a potential target in AML therapy.

CD13 is preferentially expressed on AML cells, LSC colon epithelial cells and kidney tubular epithelial cells. Targeting CD13 alone could lead to CAR T-mediated on-target/off-tumor toxicity toward human HSCs and other normal cells. A recent study showed that CAR-T cells that were bispecific for CD13 and Tim-3 exhibited reduced toxicity to human bone marrow stem cells and peripheral myeloid cells in mouse models, which highlights a promising approach for the development of effective AML CAR-T cell therapy (69). Thus, anti-human Tim-3 was identified as a potential strategy for curing AML by targeting LSCs.

### Clinical Research

Clinical trials related to Tim-3 are still limited, and the underlying mechanisms of Tim-3 in humans are still unclear. Tim-3 is highly expressed in non-M3 AML patients and might be associated with clinical characteristics and the response to induction chemotherapy in *de novo* non-M3 AML (70). Haubner et al. (58) found that Tim-3 was not only expressed on AML LSCs (78.5%) but also had 87.3% positive expression based on flow cytometry of primary AML (n = 302) samples. Dama et al. showed that patients who underwent successful treatment with selinexor and high-dose cytarabine and mitoxantrone (NCT02573363) had higher frequencies of Gal-9^+^CD34^−^ cells than patients with unsuccessful remission, with increased Tim-3 expression in these failure cases. Tim-3 expression is higher in the bone marrow than in the peripheral blood. Additionally, Gal-9/Tim-3 interaction-based treatment combined with induction chemotherapy was suggested to support complete remission for AML patients (71).

Novartis Pharmaceuticals began a clinical trial of PDR001 (a PD-1 antibody) and/or MBG453 in combination with decitabine (a DNA hypomethylating agent) in patients with AML or high-risk MDS in 2017 (NCT03066648). MBG453 is a high-affinity, humanized anti-Tim-3 IgG4 monoclonal antibody that blocks the binding of Tim-3 to PtdSer. In 2019, Novartis Pharmaceuticals began another clinical trial in which HDM201 (an MDM2 inhibitor) was combined with MBG453 or venetoclax (a BCL2 inhibitor) in patients with AML or high-risk MDS (NCT03940352). Although these clinical trials have not yet published data, the continuation of these clinical trials indicates that the Tim-3 antibody strategy for AML is attractive. There will be promising results soon.

## Future Perspectives

Tim-3 is a negative regulator of anticancer responses to vaccines and chemotherapy. Tim-3 antibodies could be a good adjuvant for vaccines, and cancer vaccines in combination with anti-Tim-3 mAbs have been identified as a novel strategy for AML therapy (65). Baghdadi et al. (67) found that combined blockade of Tim-3 and Tim-4 could augment cancer vaccine efficacy in a mouse melanoma model. Tim-3 blockade mainly stimulates antitumor effector activities *via* NK cell-dependent mechanisms. Ma et al. (66) found that Tim-3 blockade enhanced virus-specific CD8^+^ T cell responses in *L. monocytogenes*-HCV vaccine-infected DCs, and blocking Tim-3 signaling significantly improved innate and adaptive immune responses in chronic HCV-infected patients. In a previous study, Wang et al. (15) found that Tim-3 blockade could increase macrophage MHC-I expression and macrophage-mediated antigen presentation, which led to enhanced CD8^+^ T cell activation. These studies indicate that Tim-3 blockade could boost immunity and aid vaccine inoculation.

Targeting Tim-3 is similar to using one stone to kill two birds and balances the immune system in AML while killing LSCs (**Figure 1**). AML cells can escape immune surveillance (35, 36). However, little is known about the signaling mechanisms by which AML cells are exposed to immune cells and escape surveillance. On the one hand, Tim-3 is constitutively expressed on many innate and adaptive immune cells and is involved in multiple checkpoint inhibitor immunoregulatory processes (43). Targeting Tim-3 can revive the immune system in AML. On the other hand, Tim-3 is a potential functional molecule on the surface of LSCs and might shed light on how to eradicate LSCs without harming hematopoiesis (43). These findings make a Tim-3-targeted therapeutic strategy potentially beneficial for AML therapy, and such therapeutic strategies have been applied in preclinical or clinical trials.

There are still some unresolved problems. There are two possible solutions to the design of a Tim-3-targeted therapy. One is FC-dead or IgG4 monoclonal antibodies, which could inhibit Tim-3 signaling in the immune system and LSCs. The other is the use of Tim-3 CAR-T or IgG1 Tim-3 antibodies with complement-dependent cytotoxicity (CDC) and ADCC function, which could kill AML LSCs, dysfunctional immune cells with Tim-3 expression and differentiated monocytes. Elimination of dysfunctional Tim-3-expressing immune cells could be a new way to address immune tolerance. Although Tim-3 CAR-T and IgG1 antibodies could kill differentiated monocytes, they are not more toxic than chemotherapy in AML patients, and HSCs remain viable, supporting reconstitution of the immune system. Tim-3 CAR-T cells could be followed by hematopoietic stem cell transplantation (HSCT) and provide an opportunity for AML (relapse/refractory) treatment with HSCT. Due to the importance of Tim-3 in AML, the underlying mechanisms require further investigation.

Thus, it is important to examine the mechanisms of Tim-3 in types of multiple immune cells, which might be a valid therapeutic strategy for cancer immunotherapy. However, there are more studies relating to the role of Tim-3 in solid tumors than in AML (70). The mechanism of Tim-3 in AML is still not clear and needs to be further explored.

## Author Contributions

ZW and JC drafted the manuscript. ZW and LY conceived the idea, and LY supervised the process. MW and LZ helped with the opinion and discussion in clinical. All authors contributed to the article and approved the submitted version.

## Funding

This work was supported by grants from the “Major New Drug Development Project” from Ministry of Science and Technology of China (2019ZX09201002003), the State Key Program of National Natural Science of China (82030076), the National Natural Science Foundation of China (82070161, 81970151, and 81870134), the Beijing Natural Science Foundation (7202186), the Science and Technology Foundation of Shenzhen (JCYJ20200109113810154), the Shenzhen Science and Technology Investigation Project (JCYJ20190808163601776), and the Natural Science Foundation of Shenzhen University General Hospital (SUGH2020QD008).

## Conflict of Interest

The authors declare that the research was conducted in the absence of any commercial or financial relationships that could be construed as a potential conflict of interest.
